# microRNA-184 in the landscape of human malignancies: a review to roles and clinical significance

**DOI:** 10.1038/s41420-023-01718-1

**Published:** 2023-11-24

**Authors:** Mehdi Fattahi, Delsuz Rezaee, Fatemeh Fakhari, Sajad Najafi, Seyed Mohsen Aghaei-Zarch, Parisa Beyranvand, Mohammad Amin Rashidi, Saeid Bagheri-Mohammadi, Fahimeh Zamani-Rarani, Mohammad Bakhtiari, Abbas Bakhtiari, Shahab Falahi, Azra Kenarkoohi, Jamal Majidpoor, P. U. Nguyen

**Affiliations:** 1 Institute of Research and Development, Duy Tan University, Da Nang, Vietnam; 2 School of Engineering & Technology, Duy Tan University, Da Nang, Vietnam; 3 School of Allied Medical Sciences, Ilam University of Medical Sciences, Ilam, Iran; 4 Department of Toxicology and Pharmacology, School of Pharmacy, Tehran University of Medical Sciences, Tehran, Iran; 5 Department of Medical Biotechnology, School of Advanced Technologies in Medicine, Shahid Beheshti University of Medical Sciences, Tehran, Iran; 6 Cellular and Molecular Biology Research Center, Shahid Beheshti University of Medical Sciences, Tehran, Iran; 7 Department of Medical Genetics, School of Medicine, Shahid Beheshti University of Medical Sciences, Tehran, Iran; 8 Department of Molecular Genetics, National Institute of Genetic Engineering and Biotechnology (NIGEB), Tehran, Iran; 9 Student Research Committee, Department of Occupational Health and Safety, School of Public Health and Safety, Shahid Beheshti University of Medical Sciences, Tehran, Iran; 10 Department of Physiology and Neurophysiology Research Center, School of Medicine, Shahid Beheshti University of Medical Sciences, Tehran, Iran; 11 Department of Anatomical Sciences, School of Medicine, Isfahan University of Medical Sciences, Isfahan, Iran; 12 Behbahan Faculty of Medical Sciences, Behbahan, Iran; 13 Anatomical Sciences Department, Medical Faculty, Hamadan University of Medical Sciences, Hamadan, Iran; 14 Zoonotic Diseases Research Center, Ilam University of Medical Sciences, Ilam, Iran; 15 Department of Microbiology, Faculty of Medicine, Ilam University of Medical Sciences, Ilam, Iran; 16 Department of Anatomy, Faculty of Medicine, Infectious Disease Research Center, Gonabad University of Medical Sciences, Gonabad, Iran

**Keywords:** Cancer, Cancer

## Abstract

MicroRNAs (miRNAs) are a class of non-coding RNAs (ncRNAs) with a short length of 19–22 nucleotides. miRNAs are posttranscriptional regulators of gene expression involved in various biological processes like cell growth, apoptosis, and angiogenesis. miR-184 is a well-studied miRNA, for which most studies report its downregulation in cancer cells and tissues and experiments support its role as a tumor suppressor inhibiting malignant biological behaviors of cancer cells in vitro and in vivo. To exert its functions, miR-184 affects some signaling pathways involved in tumorigenesis like Wnt and β-catenin, and AKT/mTORC1 pathway, oncogenic factors (e.g., c-Myc) or apoptotic proteins, such as Bcl-2. Interestingly, clinical investigations have shown miR-184 with good performance as a prognostic/diagnostic biomarker for various cancers. Additionally, exogenous miR-184 in cell and xenograft animal studies suggest it as a therapeutic anticancer target. In this review, we outline the studies that evaluated the roles of miR-184 in tumorigenesis as well as its clinical significance.

## Facts

- miR-184 shows downregulation in a majority of cell and clinical studies of cancer
- Experimental investigations document an anticancer role for miR-184 suppressing proliferation, migration, invasion, and metastasis of tumor cells
- Ectopic expression of miR-184 demonstrates therapeutic benefits in cell and animal studies
- miR-184 can act as a diagnostic/prognostic biomarker for cancer patients

## Open questions

- Does miR-184 show antitumor role for all human cancers?
- How can be interpreted some controversial findings on the role of miR-184?
- What are the targets of miR-184 and how they can be manipulated for cancer therapy?
- Do large clinical studies support the role and significance of miR-184 in human cancers?

## Introduction

MicroRNAs (miRNAs) are a class of endogenous small (containing 19-25 nucleotides) group of non-coding RNAs (ncRNAs) that are widely found in eukaryotic cells [1]. Although, the precise roles of ncRNAs are not well elucidated, based on a developing extent of research evidence, they are suggested to function as posttranscriptional gene expression regulators [2]. In addition to transcription regulation, through interaction with other subclasses like sponging miRNAs by circular RNAs (circRNAs), interaction with RNA-binding proteins (RBPs), and translation into proteins, ncRNAs are involved in regulating the translation of protein-coding mRNAs as well as process of various RNA molecules [3, 4]. Estimations show that miRNAs may control an approximately one-third of the human genome [5]. The main mechanism of action identified for miRNAs is through degradation or translational inhibition and decreasing the stability of specific target messenger RNAs (mRNAs) via binding to their 3′ untranslated regions (UTRs) [6]. By development of high throughput technologies like reverse transcription polymerase chain reaction (qRT-PCR), chips, and miRNA profiling using the next generation sequencing (NGS), the count of identified miRNAs shows constant increasing and accordingly several databases (e.g., miRBase) are created for depositing and providing access for researchers and scientists [7–9]. A growing body of studies has indicated that miRNAs may exert critical roles in regulating various fundamental biological processes, such as inflammation, metabolism, cell growth, differentiation, development, apoptosis, and migration [10–12]. Accordingly, a large number of clinically significant diseases are related to the abnormal expression level of miRNAs, including cancer, cardiovascular diseases (heart failure), autoimmune diseases, neurodevelopmental conditions (Down syndrome, Alzheimer’s disease), asthma, frailty, liver disease (viral hepatitis), skeletal muscle, and skin disorders (psoriasis) [13–16]. Accumulating evidence has identified that an alteration in miRNAs level occurs in various human cancers [17, 18]. Therefore, miRNAs are associated with tumorigenic processes like cell growth and differentiation, angiogenesis, metastasis, and apoptosis [19]. According to the putative function of miRNAs in cancer studies, they are classified into two groups of oncogenic (oncomiRs) and tumor suppressor miRNAs. As their names suggest, oncomiRs are upregulated miRNAs in cancer cells and tissues, which functionally promote the oncogenic phenotype of tumor cells, including their proliferation as well as migratory and invasive capacities, while suppressing apoptosis [20]. Tumor suppressor miRNAs unlike the first category show downregulation in cancers and are represented with antitumor effects that negatively impact the potential of cancer development, progression and metastasis [21, 22]. The miRNA expression profile is documented for many types of cancer [5]. Accordingly, miRNAs are increasingly suggested with diagnostic, prognostic, and therapeutic targets in malignancies [23, 24]. Among a huge number of miRNAs being studied in human cancers, miRNA-184 (miR-184) has been identified as an evolutionarily conserved miRNA with abnormal expression in many malignant tumors. It is documented that miR-184 shows widely deregulation in numerous cancers with controversial functions either as an oncomiR in some cancers or dominantly a tumor suppressor miRNA [25]. Interestingly, miR-184 is also found with clinical potential in diagnosis, prediction of prognosis, and as a therapeutic target for some cancers. Thus, in this study, we discuss the roles and clinical significance of miR-184 in different types of cancers.

## miR-184ʼs biogenesis

**Fig. 1:**
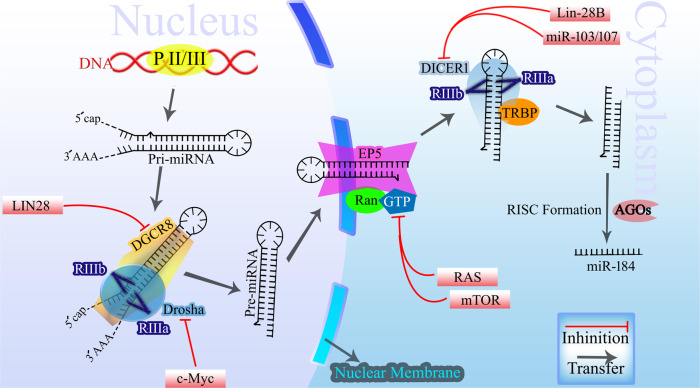
The biogenesis of miRNAs, including miR-184, and the regulating players in cancer. miR-184 is synthesized and processed according to a common mechanism known for miRNAs (details within the text). In cancer, the overexpression of LIN28, MYCN, and Oct4 inhibits DGCR8 expression. Dysregulation of c-Myc, LIN28, and DDX5 directly suppresses Drosha. Abnormalities in NPM1, RAN, and CRM1 disrupt Exportin-5. Mutations in RAS genes, dysregulation of the mTOR pathway, and anomalies in IMPDH and GART disturb GTP levels. Furthermore, dysregulation of NPM1, TPX2, CRM1 inhibit Ran, and overexpression of Lin-28B, and miR-103/107 affects nucleocytoplasmic transport and suppresses Dicer expression.

As shown in Fig. 1, the biogenesis of miR-184, same to other miRNAs, involves a series of intricate steps that culminate in the production of a fully functional miRNA molecule. This process for all miRNAs can be broadly categorized into two stages: transcriptional and posttranscriptional, each comprising multiple pivotal steps. RNA polymerase II initiates the transcription of the miRNA gene, resulting in the synthesis of a primary miRNA transcript (pri-miRNA) in the nucleus. The pri-miRNA adopts a hairpin-like structure and undergoes processing within the nucleus facilitated by the Microprocessor protein complex, which comprises the RNase III enzyme Drosha in addition to DGCR8. This complex recognizes and cleaves the hairpin structure of the pri-miRNA, liberating a precursor miRNA (pre-miRNA) with an ~70–100 nucleotides length and a stem-loop structure. After cleavage, a complex composed of exportin-5-Ran-GTP is responsible for the transportation of pre-miRNA to the cytoplasm. Upon reaching the cytoplasm, pre-miRNA transcript undergoes further processing mediated by Dicer, another RNase III enzyme. Dicer recognizes the structural characteristics of the pre-miRNA and cleaves it, generating a short double-stranded RNA duplex. This duplex consists of a mature guide strand, and a passenger strand on the opposite direction. To ensure the functional activity of miR-184, the guide strand selectively incorporates itself into the RNA-induced silencing complex (RISC). The RISC complex, composed of Argonaute proteins and associated factors, facilitates the binding of the guide strand and subsequent unwinding of the passenger strand. The unwound passenger strand is then degraded, and the guide strand remains within the RISC, poised to exert its regulatory effects [26, 27].

Within the context of cancer, numerous molecules can exert effects on the genes involved in the synthesis of miRNAs. Overexpression of LIN28 and MYCN, as well as Oct4, a transcription factor associated with pluripotency, in cancer, can impede the expression or function of DGCR8, leading to a diminished production of miRNAs [28]. Additionally, the overexpression of c-Myc and LIN28, coupled with the dysregulation of DDX5 in cancer, can directly suppress the expression of Drosha, resulting in reduced levels of miRNAs. Furthermore, activating mutations in RAS genes, dysregulation of the mTOR pathway can disturb GTP levels and nucleotide metabolism, thereby impacting the synthesis of miRNA. The overexpression of Lin-28B and the action of miR-103/107 can suppress Dicer expression, leading to a reduction in miRNA levels [29–33] (Fig. 1).

## The physiologic functions of miR-184

The encoding gene of miR-184, as a conserved, newly identified miRNA, is located on human chromosome human 15q25.1. It encodes a small transcript with 84 bp length, while no other clustered miRNAs are found near miR-184-encoding region [34]. This miRNA demonstrates uncommon properties compared to many other intergenic miRNAs [34]. It has been proved that miR-184 shows a tissue-specific expression pattern and its abnormal function may result in tissue-specific diseases [35]. In the fly *Drosophila*, miR-184 is found with involvement in numerous biological functions, particularly female germline stem cell differentiation [36]. It is also documented with crucial roles regulating the differentiation of other types of stem cells like embryonic stem (ES) cells and periodontal ligament stem cells as well as physiological functions of granulosa cells [37–39]. Particularly, its function in the regulation of cell physiologic processes like neural cells’ growth and differentiation may suggest therapeutic potential in human diseases [40]. The methyl-CpG binding protein 1 (MBD1) is a regulatory protein functions in modulating gene expression by an epigenetic mechanism based on DNA methylation. In adult neural stem/progenitor cells (aNSCs), MBD1 is shown to target several miRNAs, among them miR-184 is specifically repressed [41]. miR-184 at high levels enhanced the proliferation, while suppressed the differentiation of aNSCs. Mechanistically, miR-184 was found to exert its role through targeting Numblike (Numbl) that is discovered in brain development [41]. Moreover, miR-184 was reported to involve in the eye development, migration of keratinocytes, and corneal epithelial differentiation [42, 43]. The expression levels of miR-184 are documented to be high in the committed epidermal and corneal epithelium cells and regulated the balance between epidermal cell proliferation and differentiation [44]. A body of studies report involvement of miR-184 in the development mechanisms of human diseases, which is the subject of the current study.

## The role of miR-184 in human diseases

In 2006, expression of miR-184 was reported in a mouse model, according to which the suprabasal cells of the corneal epithelium exhibited strong expression [45]. miR-184 is identified as the miRNA with the highest expression in the cornea and lens and it can partly explain association between this miRNA dysregulation and eye diseases. In 2011, a report demonstrated that a miR-184 mutation causes familial keratoconus with cataracts [46]. In the corneal tissue, miR-184 can alter Akt and vascular endothelial growth factor (VEGF) pathways, resulting in angiostatic properties. miR-184 is shown to negatively affect angiogenesis by regulating proangiogenic secreted factors, such as VEGF. This process is vital for the functional light passing to the lens and helps the visual sense, while miR-184 is also involved in the early development of the eye [47]. Moreover, miR-184 is significantly expressed in the human retinal pigment epithelium (RPE) and its downregulation is reported in several eye conditions like age-related macular degeneration (AMD) [48]. Point mutations at miR-184 are found in association with the pathogenesis of lens/corneal dystrophy and blindness [43, 49–51]. Downregulation of this miRNA alters the levels of EZR-bound LAMP-1 protein and affects the process of phagocytosis, which further interferes with the normal function of PRE [48]. A single base mutation in miR-184 is identified in a rare genetic eye disorder called EDICT syndrome, which is presented with several ocular manifestations, such as dystrophy of epithelium cells, hypoplastic changes in iris, congenital cataract, and thinning of the stromal layer [49]. It is also reported as a contributor of secondary cataract (SC) that is developed due to differentiation of remaining lens epithelial cells after extracapsular cataract extraction. By applying negative regulatory impact on the proliferative, migratory and cell cycle progression capacities of the corneal epithelial cells, miR-184 is found to delay the corneal epithelial wound healing (CEWH) process via targeting *CDC25A*, *CARM1*, and *LASP1* genes in mice [52]. A complex, competitive RNA network, including miR-184, regulates the formation of SC in mice [53]. Moreover, miR-184 modulated Wnt signaling pathway by directly targeting frizzled-7 suggested its decreased expression can function in the pathogenesis of oxygen-induced retinopathy (OIR) via aberrant activation of Wnt signaling [54]. Ischemic stroke causes brain damage in affected patients. Various elements, such as oxidative stress and inflammation contribute to the process of ischemic stroke [55]. Evidence has shown that in rodents and humans, altered expression of many miRNAs is an important biomarker following ischemic stroke [56]. Among them, miR-184 is reported with downregulated expression following ischemic stroke in male rats. However, high levels of miR-184 can reduce subsequent brain damage [57]. Seizure preconditioning (brain exposure to a sub-threshold stimulant) can reduce the harmful effects of prolonged seizures (epileptic tolerance). The mouse model results revealed that seizure preconditioning upregulated miR-184 in the neurons, while its in vivo inhibition interfered with the protective mechanism of epileptic tolerance and resulted in neural death and brain damage after severe seizures [58]. Autism spectrum disorders (ASDs), including the fragile X syndrome and Rett syndrome are associated with synaptic plasticity defects. Both miRNA and DNA methylation pathways are involved in this process. miR-184, as a brain-specific miRNA, is discovered to exert pathogenic roles in Rett syndrome [59], which the disease is characterized by neuropsychiatric abnormalities [60]. miR-184 plays a substantial role in differentiating ES cells to some cardiac cells like mesoderm and cardiomyocytes by regulating Wnt3 expression [37]. The results of a cell model study have shown that inhibition of miR-184 suppressed inflammation and oxidative stress process, and as a result prevented apoptosis in cardiomyocytes [61]. miR-184 also constitutes an abundant number of miRNAs in pancreatic β-cells, which affects the cellular pathways of insulin secretion and impacts glucose metabolism as an insulin release suppressor [62]. Those findings have suggested miR-184 as a crucial role player in fine-tuning and regulation of pancreatic β-cell function. Downregulation of miR-184 is documented in human pancreatic islets of patients with type 2 diabetes relative to healthy donors [63]. Importantly, miR-184 inhibition in human β-cells protected them against palmitate-induced apoptosis as well as metabolic and inflammatory stress conditions. Those effects were found to be exerted through targeting the CREB transcriptional coactivator 1 (CRTC1), while CRTC1 overexpression showed reverse effects compared to miR-184 inhibition. Importantly, the transcription factor NKX6.1 with specific expression in β-cells regulated miR-184 expression via binding to its promoter sequence in human and in murine β-cells [63]. This miRNA is also found to play a role in the pathogenesis of cyanotic congenital heart diseases (CHD) via regulating the proliferation and apoptosis of cardiomyocytes [64]. Furthermore, miR-184 is found to function in the development of several other human diseases like Kawasaki disease [65], Recurrent spontaneous abortion (RSA) [66], and sepsis [67], while evaluation of its expression levels has suggested it as a potential diagnostic and predictive biomarker for conditions like polycystic ovary syndrome (PCOS) [68], and Anderson-Fabry disease (AFD) [69].

## miR-184 in cancers

The carcinogenesis process is composed of several steps involving two classes of genes, protooncogenes and tumor suppressor genes [70]. Genetic alterations drive the normal cells to progress from the pre-malignant initiative status to malignant phenotype [71]. According to the global cancer statistics 2020, 19.3 million new cases in addition to 10.0 million deaths were reported for 2020 [72]. Early detection of cancer patients might improve the survival of patients by providing real-time access to therapies. Imaging techniques, laboratory tests, and tumor biomarkers constitute the main diagnostic approaches used for cancer detection, which still lack sufficient performance for real-time screening and diagnosis of cancer patients [73]. Current therapeutic strategies include chemotherapy, and radiation therapy, in addition to immunotherapy as a novel category of anticancer therapy [74, 75]. Although considerable achievements have been in improving the efficacy of anticancer therapeutics like hopeful findings reported for anticancer vaccines and immune checkpoint inhibitors (ICIs) [76, 77]; however, resistance to anticancer agents and late diagnosis might negatively affect survival among patients [78]. Resistance to anticancer therapies is mainly considered due to the tumor microenvironment (TME) and existence of some elements like cancer stem cells (CSCs) that in turn can affect the survival of cancer patients [79].

Several years of research have demonstrated that miRNAs might play crucial roles in all known physiologic activities similar to normal cells as well as pathogenic mechanisms in cancer cells, including cell growth and death, aggressiveness behaviors, metabolism, and survival [80]. Dysregulation of miRNA expression might affect the development, progression, and dissemination of human tumors, which are mechanistically conducted by regulating the expression of specific target genes. Both oncomiRs and tumor suppressor miRNAs are reported with substantial functions in regulating the malignant behaviors of tumor cells. Enhanced expression of oncomiRs or downregulation of tumor suppressor miRNAs suggests therapeutic potential, particularly in cancers. To achieve that, both miRNA mimics and antagonists have emerged as potential therapeutic agents that act by interfering with various cancer molecular pathways [80]. Some potential therapeutics include antisense oligonucleotide (ASO) strategy to inhibit the function of oncomiRs, exogenous miRNAs for augmentation of the tumor suppressor miRNAs expression, and artificial miRNA for targeting malignant tumor phenotype-related genes [71]. Also, the clustered regulatory interspaced short palindromic repeat (CRISPR)-associated nucleases (Cas) technology that uses a novel technology for manipulating genomes of various organisms by delivering Cas9 protein and a guide RNA to the target cells [81] may suggest potential for targeting and inhibition of oncomiRs [82]. Importantly, miRNAs have been found in increasing experimental data as biomarker candidates for various human diseases, including cancer [83]. This is not limited to miRNAs, while other classes of ncRNAs, including long transcripts (lncRNAs) and circRNAs, are also extensively studied as biomarkers to help diagnosis and prediction of prognosis among cancer patients [84–90]. Detection of miRNAs in the serum and other biological fluids (known as liquid biopsy) of cancer patients potentially can help non-invasive diagnosis of different cancers [91]. miR-184 is mainly reported with a tumor suppressor role suppressing the proliferation, migration, and invasion and induces apoptosis of cancer cells. Mechanistically, it is documented acting through targeting various genes to exert a suppressing role on the malignant behavior of cancer cells. Impacting some signaling pathways like Wnt and β-catenin, and AKT/mTORC1 pathway, oncogenic factors (e.g., c-Myc) or apoptotic proteins, such as Bcl-2 can partly explain some mechanisms through which miR-184 may exert its role as a tumor suppressor (Table 1). Xenograft animal studies also have supported the inhibiting impact of miR-184 on tumor growth and metastasis. Interestingly, aberrant expression of miR-184 is revealed with a prognostic and diagnostic performance for a number of human cancers.

**Table 1:** A summary to studies reported the significance of miR-184 in human cancers and their clinical significance.

Cancer	Expression change	Investigation method	Population	Pathological Specimen	Target (s)	Significance	Clinical potential	Refs.
Lung cancer	Down	miR-184 transfection, PCR, Luciferase reporter assay, ChIP assay	Patients, Cell lines	59 NSCLC patients, SiHa, and C33A cells	Bcl-2	miR-184 overexpression conferred resistance to cisplatin in cell lines and also predicted unfavorable response to chemotherapy among NSCLC patients.	Prognostic and therapeutic	[97]
	Down	Boyden chamber assay, qRT-PCR	Patients, Cell lines	124 NSCLC tissue biopsies	CDC25A and c-Myc	Inhibition of NSCLC cell proliferation and invasion	Prognostic and therapeutic	[98]
	Down	Immunoblotting, microarray and qRT-PCR	SCLC Patients, Cell lines	72 Serum and tissue biopsy	β-catenin signaling and EPAS1	Suppression of SCLC metastasis and invasion in vitro	Prognostic and therapeutic	[100]
Endometrial carcinoma	Down	Transfection, Transwell invasion assay, qRT-PCR, dual luciferase reporter assay	EC tissues, Cell lines	44 fresh EC tissue specimens	CDC25A	Suppression of growth and invasion of EC cells	Therapeutic	[112]
Prostate cancer	Down	qRT-PCR, bioinformatics, and Dual-luciferase reporter gene assay	Tumor tissues, Cell lines	499 tumor tissues	DLX1	Inhibition of cell propagation, migration and invasion	Therapeutic	[117]
Breast cancer	Down	qRT-PCR, microarray, bioinformatics, WB, and Luciferase reporter assay	Orthotopic xenografts, cell lines	–	AKT/mTORC1 pathway	Suppression of tumor growth in vitro and in vivo	Prognostic and therapeutic	[122]
	Down	qRT-PCR, CCK-8, BrdU, Transwell, and wound healing assay	Cell lines	Human BC cell lines (MCF-7 and BT-474)	Bcl-2	miR-184 and tripterine synergistically restrained the viability, proliferation, and invasion of BC cells.	Therapeutic	[121]
Colorectal cancer	Down	qRT-PCR, CCK-8, Transwell assay, WB analysis, bioinformatics, and luciferase reporter assay	Tumor tissues, Cell lines	32 CRC tissues	IGF-1R	Inhibition of proliferation, migration, and invasion of CRC cells	Therapeutic	[127]
Gastric cancer	Down	Transfection, qRT-PCR, Transwell assay, WB, and bioinformatics analysis	Cell lines	MGC803 GC cells and GES1 and AGS normal cell lines	Stanniocalcin 2 (STC2)	Inhibition of proliferation and invasion of GC cells	Therapeutic	[132]
Hepatocellular carcinoma	Up	In silico analyses and luciferase reporter assay	Cell lines	HepG2 HCC cells	INPPL1 and caspase 3/7	Enchantment of cellular proliferation and inhibition of apoptosis	Therapeutic	[137]
HNSCC	Up	qRT-PCR	Tumor tissues, Cell lines	20 human samples of tongue SCC	c-Myc	Enhancement of cell proliferation and inhibition of apoptosis	Prognostic and therapeutic	[140]
NPC	Down	Microarray, WB, qRT-PCR, Immunohistochemistry, dual luciferase reporter assay	Cell lines, tumor xenograft model	5–8 F, 6-10B, HNE1, SUNE1, CNE-1, and CNE-2 cell lines	Notch2	Inhibition of cell invasion and metastasis in vitro and in vivo	Therapeutic	[153]
Glioma	Down	Bioinformatics, microarray, immunohistochemistry, WB, colony formation assay, and flow cytometry	Tumor tissues, Cell lines, tumor xenograft model	10 glioma tissues and 5 normal brain tissues	SND1	Inhibition of cell growth and invasion in vitro and in vivo	Therapeutic	[107]
	Up	transfer miR-184 by lipidosomes, qRT-PCR, WB, MTT, and plate cloning	Tumor tissues, Cell lines	Glioma tissue samples	FOXO3	Enhancement of cell proliferation and colony formation	Therapeutic	[108]
	Down	qRT-PCR, flow cytometry, wound healing and invasion assay, WB, immunohistochemistry	Tumor tissues, Cell lines, tumor xenograft model	81 glioma samples	TNFAIP2	Suppression of cell proliferation, migration, and invasion and induction of apoptosis as well as cell cycle arrest in vitro and tumor growth in vivo	Therapeutic	[35]
Osteosarcoma	Up	Bioinformatics, qRT-PCR, WB, Annexin V apoptosis detection, and luciferase reporter assay	Cell lines	U-2 OS and MG-63	BCL2L1	miR-184 upregulation led to poor response to drug therapy	Therapeutic	[145]
	Up	Transfection, qRT-PCR, MTT and Transwell assays, WB	Cell lines, tumor xenograft model	U-2OS cells and 143B cells	Wnt and β-catenin	Inhibition of cell proliferation and invasion in vitro and tumor metastasis in vivo upon miR-184 overexpression	Therapeutic	[25]
	Up	qRT-PCR, CCK-8 assay, wound healing assay, and flow cytometry	Tumor tissues, Cell lines	18 OS patients, SOSP-9607 cells	–	miR-184 inhibition may suppress the proliferation, and migration, and induce the apoptosis of OS cells	Therapeutic	[148]
Ovarian cancer	Down	qRT-PCR, WB	Tumor tissues, Cell lines	80 EOC tissues	Bcl-2, Bax, caspase-3 and inflammatory factors	Inhibition of cell proliferation and induction of apoptosis of EOC cells and suppression of inflammatory cytokines	Prognostic and diagnostic	[156]
Renal cell carcinoma	–	Transfection, qRT-PCR, Cell proliferation, and apoptosis assays	Cell lines	786-o and ACHN	–	Inhibition of cell proliferation and migration and induction of apoptosis of RCC cells	Therapeutic	[174]
Retinoblastoma	Up	Cell Transfection, luciferase Assay, qRT-PCR, immunofluorescence, WB, flowcytometry	Tumor tissues, Cell lines	15 paraffin-embedded human RB tissues	SLC7A5	Inhibition of cell proliferation, migration, and invasion and enhancement of chemosensitivity	Therapeutic	[161]
Pancreatic adenocarcinoma	Down	Transfection, qRT-PCR, flow cytometry, Annexin V apoptosis Analysis, WB	Tumor tissues, Cell lines	120 PDAC tissues	C-Myc, C-Jun, and Bcl-2	Inhibition of proliferation and promotion of apoptosis of PDAC cells	Prognostic	[175]
Clear cell renal cell carcinoma	Down	qRT-PCR, WB, CCK8 proliferation assay, Transwell assay, Annexin V apoptosis Analysis, luciferase reporter assay	Cell lines	A-498 and 786-O cell lines	NUS1	Suppression of cell proliferation, and invasion and induction of apoptosis	Prognostic and therapeutic	[157]
Melanoma	Up	qRT-PCR, tube formation assay	Cell lines	C8161 and C81-61 cells	–	Inhibition of cell proliferation, invasion, and tube formation	Therapeutic	[158]

WB: western blotting, MTT: 2,5-diphenyl-2H-tetrazolium bromide; CCK-8: Cell Counting Kit-8; qRT-PCR: quantitative reverse transcription polymerase chain reaction; PDAC: pancreatic ductal adenocarcinoma; CRC: colorectal cancer; BCL2L1: Bcl-2-like protein 1; SCLC: small-cell lung cancer; EPAS1: endothelial PAS domain protein 1; EC: endometrial carcinoma; NPC: nasopharyngeal carcinoma; EOC: epithelial ovarian cancer.

## Lung cancer

Lung cancer is responsible for the highest number of cancer-related mortalities globally [92]. Non-small-cell lung cancer (NSCLC) is one category of lung cancer with a poor prognosis and chemotherapy resistance [93]. Modern targeted therapies for NSCLC are more efficient and specific in comparison with conventional radio-chemotherapy [94]. Recent studies indicate that miRNAs expression profile is different between the resistant and sensitive tumors under the epidermal growth factor receptor tyrosine kinase inhibitors (EGFR-TKIs) therapy [92]. Results of a case-control study revealed that miR-184 expression was considerably higher in patients with NSCLC compared to those with benign lung diseases. It was also confirmed that after 3 years of follow-up, the levels of exosomal miR-184 in serum specimens of the NSCLC surviving patients were considerably lower compared to non-surviving group [95]. Bcl-2 family of apoptosis-regulating factors are already shown to affect the survival of NSCLC cells [96]. miR-184 downregulation by E6 oncoprotein can confer chemoresistance to cisplatin and negatively affect therapeutic responses of NSCLC patients via enhancing Bcl-2 expression [97]. In this study, Tung et al. assessed the expression level of miR-184 in the cervical cancer SiHa cells and human papillomavirus (HPV) 16-positive TL-1 cells. Results showed that miR-184 was downregulated in TL-1 and SiHa cells compared to controls, and miR-184 regulated the proliferation and survival of those cells through modulating the c-Myc and Bcl-2 expression. Also, they discovered that expression levels of Bcl-2 and miR184 was high in E6-positive tumor samples than in E6-negative tumor samples, which finally it can be concluded that the presence of E6 oncoprotein reduced miR-184 expression, while this miRNA affected cell growth and apoptosis via exerting a suppressing impact on the target genes [97]. miR-184 is documented to act as a tumor suppressor with inhibitory effects on cell survival and invasive capability of NSCLC cells by modulating the expression of CDC25A and c-Myc expression. Interestingly, miR-184 downregulation was suggested as a contributor to unfavorable survival among NSCLC patients [98]. Small-cell lung cell (SCLC), as a highly malignant type of neuroendocrine tumor, constitutes 15–20% of lung cancer cases and has poor clinical prognosis due to fatal metastasis [99]. Results of a study revealed that miR-184 suppressed migration and invasion of NSCLC cells functioned as a tumor suppressor. miR-184 could significantly attenuate metastasis of SCLC through participating in β-catenin signaling and downregulating the endothelial PAS domain protein 1 (EPAS1), which is a transcription factor functioning in various tumors including SCLC [100]. Moreover, miR-184 is documented to be involved in inhibiting the proliferation and epithelial-to-mesenchymal transition (EMT) process of airway epithelial cells contributing to suppressing the pathogenesis of idiopathic pulmonary fibrosis (IPF) [101].

## Glioma

Glioma is the most malignant and common form of primary brain tumors in adults with an extremely bad prognosis of approximately one year [102]. The aggressive progression and invasive nature of glioma cells, facilitate the recurrence of the disease after therapies of surgery, chemotherapy, or radiation [103–105]. Recent studies provided evidence that cellular and molecular biological processes including abnormal gene expression and regulation of factors involved in tumor growth, play a significant role in glioma [106]. Thus, more attention has been paid to biological target therapy of glioma. In glioma, the expression of a handful of miRNAs, such as miR-21, miR-145, miR-940, and miR-101 are reported with downregulation while a number of other miRNAs like miR-191, miR-640, and miR-155-3p are upregulated in tumor tissues and cell lines [72]. Studies report miR-184 downregulation both in cell and tissue investigations. Exogenous expression of miR-184 decreased the proliferation and invasion of glioma cells by regulating SND1 expression. SND1 is known to be upregulated in human glioma with a regulatory role in glioma progression. Thus, miR-184/SND1 axis can be used as a target for diagnosis and therapy of malignant glioma [107]. Another study indicated that proliferation of glioma cells can be promoted by miR-184 overexpression through regulating FOXO3 [108]. Cheng et al. conducted a study, which demonstrated that miR-184 inhibited glioma progression through targeting TNFAIP2 expression and affecting its translation in glioma [35]. Results of a clinical experiment showed that miR-184 was overexpressed in glioma tissue in comparison to normal brain tissue [109]. Functional analyses revealed that miR-184 was suggested to promote the occurrence and development of gliomas. Furthermore, in higher pathological grades of glioma, miR-184 expression increased, but it did not show any correlation with the pathological type of glioma cells. However, a negative correlation was found between miR-184 expression and survival time of patients [109]. Collectively, in addition to witness supported the tumor suppressor role of miR-184, these findings suggested this miRNA as a potential prognostic and diagnostic biomarker for and a therapeutic target for glioma patients. This conclusion; however, is not supported at least by a study indicating upregulation of miR-184 and its oncogenic role enhanced the malignant phenotype and activities of A172 cells [110].

## Endometrial cancer

Endometrial cancer (EC) is a common gynecologic malignancy in females in developed countries [111]. A recent study provides evidence that decreased miR-184 expression inhibited EC cell proliferation and invasion by targeting CDC25A-dependent Notch pathway [112]. It has been also suggested that miR-184 expression correlated with lymph node metastasis and survival of EC patients [113]. miRNA expression profile was used to predict the lymph node metastasis in a group of female patients diagnosed with grade 1-2 EC. Among dysregulated miRNAs, miR-184 showed downregulation in primary tumor specimen retrieved from women with lymph node metastases (positive LN) relative to those samples from patients with negative LN [113]. Additionally, miR-184 downregulation is also reported to correlate with pelvic LN metastasis and/or recurrence among EC patients [114].

## Prostate cancer

Prostate cancer (PC) is the most commonly diagnosed cancer among men that is responsible for 27% of newly diagnosed cases and 11% of all cancer‑related deaths worldwide [115]. DLX1 is a protein that binds to β‑catenin and enhances viability and migratory capacities of cancer cells through activation of the β‑catenin/TCF signaling pathway [116]. miR‑184 is reported with downregulated expression in PC tissues and cell lines (C4-2, PC-3, Du145, and LNCaP) relative to normal prostate tissues and RWPE-2 cell lines, respectively [117]. miR-184 overexpression suppressed the growth and malignant activities of PC cells, while exogenous expression of DLX1 showed reverse impact. Mechanistically, miR-184 was found on bioinformatic investigations and dual luciferase reporter assay to directly target DLX1. This study suggested miR-184 as a tumor suppressor miRNA and a target for finding a molecular-based therapy for PC patients.

## Breast cancer

**Fig. 2:**
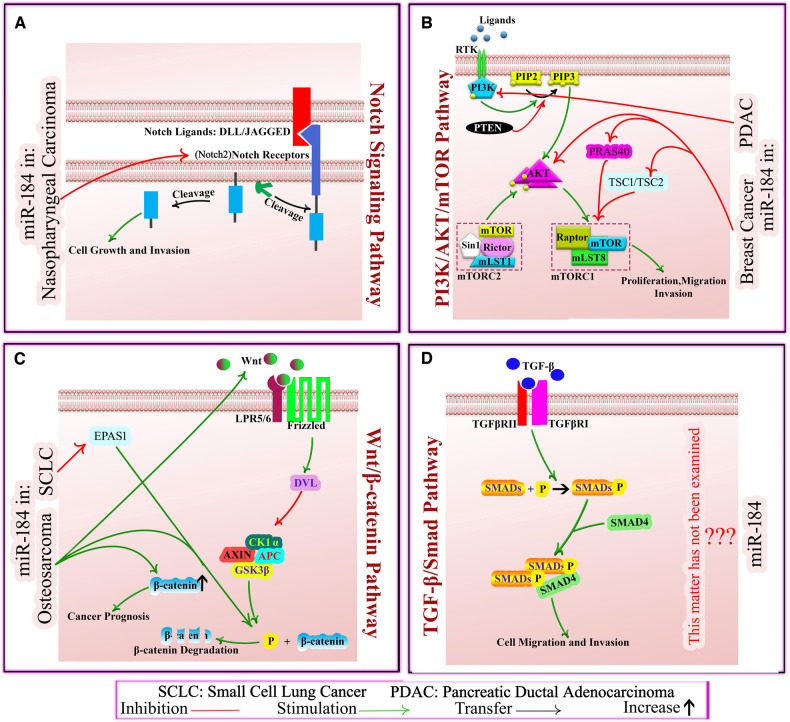
The mechanistic impact of miR-184 on signaling pathways in some cancers. **A** Notch Signaling Pathway. The binding of DLL or JAGGED to Notch receptors leads to the liberation of the NICD, which is subsequently translocated to the nucleus where it interacts with transcription factors. In Nasopharyngeal Carcinoma, miR-184 suppresses Notch2 receptor. **B** PI3K/AKT/mTOR Pathway: PI3K generates phosphatidylinositol (3,4,5)-trisphosphate (PIP3), which activates AKT and it directly targets mTOR, existing in mTORC1 and mTORC2 complexes. PTEN functions by dephosphorylating PIP3. In pancreatic ductal adenocarcinoma, miR-184 directly targets PI3K and in breast cancer, it may target mTORC1 inhibitors PRAS40 and TSC2 to eventually suppress AKT pathway. **C** Wnt/β-catenin pathway: In the absence of Wnt ligands, β-catenin is targeted for degradation by a complex comprising Axin, APC, GSK-3β, and CK1. Wnt binding to Frizzled receptors activates the LRP5/6 co-receptor, inhibiting the degrading complex and allows β-catenin to accumulate. In endometrial cancer, miR-184 inhibits Wnt, Frizzled, and DVL, whereas in cervical cancer, it downregulates Wnt. In small-cell lung cancer, miR-184 inhibits EPAS1, β-catenin phosphorylation enhancer. In osteosarcoma, miR-184 increases Wnt and enhances β-catenin levels and phosphorylation status. **D** TGF-B/Smad pathway: Ligands bind to TGF-B receptors, activating the receptor complex and phosphorylating R-Smads, which then develop complexes with Smad4, translocating into the nucleus to regulate gene expression. The effect of miR-184 on this pathway is unclear. DLL delta-like ligand, PI3K phosphoinositide 3-kinase, PIP3 phosphatidylinositol (3,4,5)-trisphosphate, mTOR mammalian target of rapamycin, mTORC mammalian target of rapamycin complexes, PTEN phosphatase and tensin homolog, PRAS40 proline-rich Akt substrate of 40 kDa, EPAS1 endothelial PAS domain protein 1, TGF-B transforming growth factor-beta.

Breast cancer (BC) is the most frequent malignancy for women and a high incidence globally, with an estimated 2,300,000 cases in addition to 685,000 deaths in 2020, which is predicted to reach 4,400,000 lives in 2070 [118]. Based on the tumor type and the clinicopathological stage, monotherapy or combinational therapy using conventional agents is recommended for BC patients [119]. According to various studies, many miRNAs are effective in regulation of initiation and progression in BC subtypes by mediating vital cellular processes, including cell proliferation, differentiation, and apoptosis [120]. Studies indicated that miR-184 is a tumor suppressor, inhibiting cell growth, self-renewal, and modulating the development of metastatic lesions to function in the BC pathogenesis [121, 122]. Phua et al. evaluated the role of miR-184 on tumorigenesis and distant metastasis in the orthotopic xenografts of BC cells. For this purpose, they investigated the expression of miR-184 and gene targets by qRT-PCR, microarray, in situ assessments, and luciferase reporter assay. To determine the miR-184 function, they transfected miR-184 into MDA-MB-436 and HS578T BC cells. Results showed that miR-184 overexpression suppressed the proliferative and self-renewal capacities of the control cells, delayed primary tumor development, and attenuated metastatic potential of BC cell lines. Findings also demonstrated that miR-184 suppressed the AKT/mTORC1 pathway, which functions in tumor invasion and metastasis. miR-184 is also reported to may target PRAS40 and TSC2, mTORC1 inhibitors, and it can suppress AKT2, in breast cancer [122] (see Fig. 2). In another study, Wang et al. evaluated the influence of miR-184 and tripterine on BC progression. They assessed the impact of miR-184 and tripterine on the human BC cell lines (MCF-7 and BT-474) using BrdU, CCK-8, Transwell, and wound healing investigations. This research discovered that cells’ viability, proliferation, migration, and invasion capacity were attenuated in BC cell lines treated with miR-184 and tripterine, while those agents had a synergistic effect in the development of BC [121]. Studies already have demonstrated that tripterine acts effectively against many types of cancer [121, 123, 124].

## Colorectal cancer

**Fig. 3:**
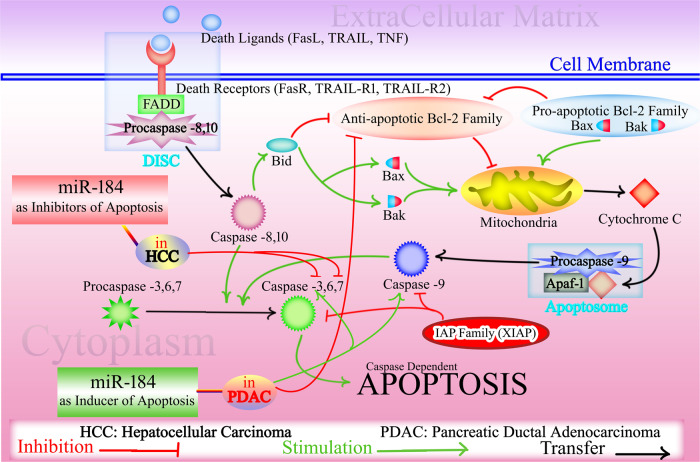
miR-184 and gastrointestinal cancers. In colorectal cancer, miR-184 can act as a tumor suppressor by directly targeting IGF-1R. In gastric cancer, miR-184 inhibits STC2, thereby suppressing cell proliferation, invasion, and metastasis. However, there is controversy surrounding the role of circ_0021087, which has been reported to exert a tumor suppressor function in gastric cancer by targeting miR-184 and upregulating FOSB. In hepatocellular carcinoma, miR-184 targets INPPL1 and caspase 3/7, leading to the inhibition of cellular proliferation and promotion of apoptosis, respectively. Additionally, miR-184 inhibits C-Myc, C-Jun, and Bcl-2, contributing to the suppression of cell growth and apoptosis induction in pancreatic adenocarcinoma. STC2 stanniocalcin 2, IGF-1R insulin-like growth factor 1 receptor, INPPL1 inositol polyphosphate phosphatase-like 1.

Colorectal cancer (CRC) is already known as the third most common malignancy and also the third cause of cancer-related deaths in both sexes accounting for 10% of all newly diagnosed cases of cancers and 9.4% of cancer mortality [72]. Numerous factors and molecular mechanisms, including genetic background and environmental agents, are involved in CRC progression [125]. miRNAs have a significant role in the initiation and development of CRC (tumor development, progression, and metastasis) [126]. As shown in Fig. 3, miR-184 can act as a tumor suppressor in CRC by directly targeting insulin-like growth factor 1 receptor (IGF-1R) [127]. Expression of IGF-1R, a transmembrane tyrosine kinase receptor of the insulin receptor family, is related to a malignant phenotype, tumor progression, chemoresistance, and unfavorable survival in CRC [128]. To analyze the function of miR-184 in CRC, Wu et al. transfected human CRC cell lines (HCT116, HT29, and SW620) and human normal colon epithelium cell line with miR-184 mimic and then evaluated the impact of mi-184 expression on the aggressive phenotype of those cells [127]. Analysis of tested groups showed that miR-184 was downregulated in CRC tissues and cell lines. Following overexpression of miR-184, 2,5-diphenyl-2H-tetrazolium bromide (MTT) and Transwell assays revealed that proliferation, migration, and invasion of CRC cells were inhibited in vitro. To understand the precise mechanism of miR-184 in CRC, western blotting (WB) analysis demonstrated that the expression of IGF-1R protein was significantly diminished in miR-184-overexpressing HCT116 and SW620 cells relative to control cells. The authors concluded that miR-184 inhibited aggressive phenotype of CRC cells by targeting IGF-1R [127].

## Gastric cancer

Gastric cancer (GC) is among the most common types of cancer, which causes high mortality worldwide [72]. The abnormal expression of miRNA could significantly affect biological characteristics of GC like cell growth, migration, invasion, and apoptosis [129]. The clinical significance of Stanniocalcin 2 (STC2) in GC was discovered by several studies [130–132]. STC2 is a glycoprotein with high expression in various tumor cells. The expression level of STC2 is upregulated in gastric tissues, and STC2 as an independent factor in cancer elevates lymphatic metastasis and venous invasion in GC [130, 133]. Qiao et al. evaluated miR-184 expression levels in MGC803 GC cell lines and human normal gastric epithelial cell lines. They also investigated the role of miR-184 as a tumor suppressor in blocking GC progression through targeting STC2, as shown in Fig. 3 [132]. The results showed downregulation of miR-184 and STC2 upregulation in GC cell lines. Ectopic expression of miR-184 in MGC803 cells significantly inhibited the expression of STC2. Consistently, STC2 inhibition suppressed the growth and invasion of GC cells indicating that miR-184 may play a tumor suppressor role [132]. Inconsistent with this study, Yu et al. reported that miR-184 reversed the inhibitory role of circ_0021087 on proliferation, EMT, migration, and invasion of GC cells [134]. Circ_0021087 exerted a tumor suppressor role in GC through targeting miR-184 and upregulating FOSB (Fig. 3). Opposite results mandate further investigations to shed light on the precise mechanism and therapeutic significance.

## Hepatocellular carcinoma

**Fig. 4:**
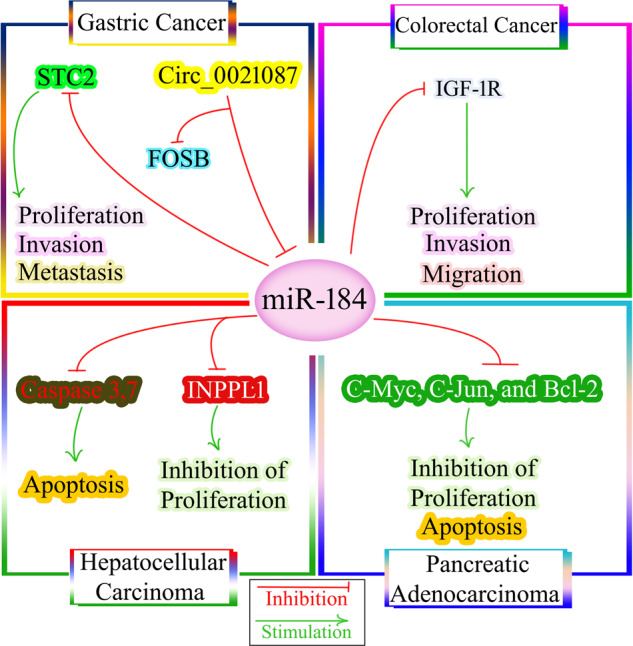
The effects of miR-184 on apoptotic pathways. The extrinsic pathway of apoptosis starts by the binding of death ligands (TNF, FasL, and TRAIL) to corresponding receptors, causing recruitment of FADD to the intracellular domain of the death receptor. This triggers the formation of DISC, which itself in a series of reactions activates initial caspase-8 or -10, and then activation of effector caspases-3, -6, and -7 leading to cell death. Caspase-8 or caspase-10 can activate Bid that in turn causes activation of pro-apoptotic proteins Bax and Bak while inhibiting anti-apoptotic members of the Bcl-2 family. In the intrinsic pathway, cellular stress signals trigger conformational changes in Bax and Bak, resulting in release of cytochrome c into the cytoplasm, where it forms the apoptosome complex with Apaf-1 and dATP. Apoptosome causes serial activation of caspases-9, -3 and -7 to initiate cell death. In cancers, miR-184 exhibits dual effects on apoptosis. It acts as an inhibitor of apoptosis by downregulating caspase-3, and caspase-6, in hepatocellular carcinoma. Conversely, in pancreatic ductal adenocarcinoma, miR-184 induces apoptosis by inhibiting anti-apoptotic Bcl-2 and increasing caspase-3 and caspace 9. TNF tumor necrosis factor, FasL Fas ligand, TRAIL TNF-related apoptosis-inducing ligand, FADD Fas-associated death domain, DISC Death-inducing signaling complex, Bid BH3-interacting domain death agonist, Bcl-2 B-cell lymphoma 2, Apaf-1 Apoptotic protease-activating factor 1, dATP Deoxyadenosine triphosphate, TRAIL-R TNF-related apoptosis-inducing ligand receptor 1, XIAP X-linked inhibitor of apoptosis.

Hepatocellular carcinoma (HCC) constitutes a majority (75–85%) of primary liver cancer cases [72]. Serum miRNAs are suggested as biomarkers for the early diagnosis and prediction of prognosis of HCC patients [135]. Among various miRNAs, miR-184 is considered as a candidate tumor suppressor inhibiting the development and progression of HCC [136, 137]. In a study by the Gao et al., the effect of miR-184 inhibition on HCC development and progression was evaluated in HepG2 cells [137]. Bioinformatics and luciferase reporter assay identified inositol polyphosphate phosphatase-like 1 (*INPPL1*) as the target of miR-184. Results revealed that the upregulation of miR-184 expression was significant in HCC tissues compared to normal tissue. Furthermore, expression analysis demonstrated a reverse relationship between INPPL1 and miR184 in HCC cells. For elucidating the role of miR-184 on apoptosis, researchers investigated the impact of anti-miR-184 on caspase 3/7 activity in HepG2 cells. Caspase 3/7 activity in anti-miR-184-treated HepG2 cells was higher than untreated cells, which they concluded that miR-184 silencing induced HepG2 apoptosis by caspase 3/7. Therefore miR-184 silencing can induce HepG2 apoptosis by caspase 3/7 and inhibit cellular proliferation by INPPL1, as shown in Fig. 3 and Fig. 4 [137].

## Head and neck squamous cell carcinoma

Head and neck squamous cell carcinoma (HNSCC) is a malignancy with high rate of incidence and mortality that occurs in oral, oropharynx, larynx, or hypopharynx [138]. Aberrant expression of miRNAs is indicated in HNSCC specimens compared with the non-malignant samples [139]. Wong et al. evaluated the plasma levels of miR-184 in the tongue squamous cell carcinoma (SCC) tissues. miR-184 expression was significantly higher in isolated tongue SCC tissues compared with control group. Also, suppressing miR-184 induced apoptosis in studied cell lines (Cal27 and HN21B and HN96). After transfection of miR-184 inhibitor into tongue SCC cell lines, a decrease in proliferation rate and downregulation of c-Myc was found. Those results demonstrated that miR-184 could act as an oncogenic factor through inhibition of apoptosis and promotion of cell proliferation in tongue SCC [140].

## Osteosarcoma

Osteosarcoma (OS) is a primary malignant cancer of bone structures that is mostly seen in children and younger adults [141]. A handful of ncRNAs including miRNAs as regulatory players are linked to tumor initiation, invasion, occurrence, and metastasis of OS [142, 143]. The impact of miR-184 in enhancing the proliferation and invasion of OS is already documented [144]. In the research by Lin et al., the role of miR-184 as a mediator of chemoresistance was investigated in OS [145]. For this purpose, miR-184 expression was evaluated following transfection of miR-184 agomir or miR-184 antagomir into OS cell lines U-2 OS and MG-63 under doxorubicin treatment. They found that miR-184 expression in transfected cells was time-dependent and inducible by doxorubicin. The luciferase reporter assay detected BCL2L1 as the direct target of miR-184. Also, in doxorubicin-treated OS cells, overexpression of miR-184 caused significant elevation in BCL2L1 expression. Ectopic expression of miR-184 agomir and miR-184 antagomir following transfection caused a decrease and promotion of doxorubicin-induced cell apoptosis of OS cells, respectively. These results showed upregulation of miR-184 occurred in doxorubicin-treated OS patients, and increasing expression of miR-184 by targeting BCL2L1 caused doxorubicin resistance in OS cells [145]. The Wnt/β-catenin pathway regulates cell renewal, cell proliferation, and differentiation in OS cells through intercellular communication by extracellular signals [146, 147]. miR-184 is already identified as one of the important miRNAs functioning in regulating the Wnt/β-catenin signaling pathway [11]. Du et al. evaluated the impact and the associated mechanism of miR-184 on the proliferation, invasion and metastasis of OS cells using in vitro and in vivo experiments [25]. Cell studies revealed upregulation of miR-184 and Wnt/β-catenin, and an increase in the proliferative and invasive potentials of miR-184-overexpressing U-2OS and 143B cells relative to un-transfected cells. In the xenograft animal study, tumor model was established in the mic. Results revealed that significant upregulation of miR-184 and Wnt and increase in phosphorylated β-catenin level. Also, miR-184 could impact the OS development and metastasis through modulating the Wnt/β-catenin signaling pathway [25]. Consistently, upregulation of miR-184 and its impact on the aggressive phenotype of SOSP-9607 OS cells is documented in another study by Tao et al. [148].

## Nasopharyngeal carcinoma

Nasopharyngeal carcinoma (NPC) is a rare type of head and neck malignant and is the most common in Southeast Asia and southern China [149]. Evidence has demonstrated that miRNAs via regulating target gene expression are associated with malignant progression of NPC [150]. The programmed cell death 4 (PDCD4), a tumor suppressor with downregulation in various tumor types, induces cell apoptosis and inhibits the aggressive phenotype of cancer cells [151]. Inhibition of BCL2 and C-MYC translation by PDCD4 suppressed cell growth and survival in NPC [152]. Zhen et al. revealed that PDCD4 protein expression was significantly reduced in NPC samples. PDCD4 suppressed the cell proliferation and cell survival in NPC through regulating the PI3K/AKT and JNK/C-Jun pathways by directly targeting C-MYC, BCL-2, and the oncogenic transcription factor C-JUN. They also showed miR-184 directly targeted the BCL2 and C-MYC and affected cell proliferation and survival in NPC [152]. In another study, Zhu et al. found downregulation of miR-184 in NPC cell lines. miR-184 suppressed the aggressive phenotype of NPC cells via regulation of EMT process and targeting Notch2 [153]. The EMT process is already known to play crucial roles during cancer progression and permits solid tumors to become more malignant [154], and thus miR-184 may function in the pathogenesis of NPC by regulating EMT.

## Ovarian cancer

Ovarian cancer is among the most prevalent causes of cancer-related deaths in female, with over 230,000 new cases and 140,000 deaths yearly [155]. Abnormal miRNAs expression is associated with ovarian cancer and can be employed as biomarkers for prediction of therapy outcomes in ovarian cancer. miR-184 is shown to downregulate in epithelial ovarian cancer (EOC) in evaluations using a high-throughput microarray study compared with immortalized ovarian surface epithelium (IOSE) cell lines. Qin et al. analyzed the expression of miR-184 in clinical EOC tissues and EOC cell lines, also the role of miR-184 in the proliferation, apoptosis, and inflammation was investigated [156]. miR‑184 expression was found to be significantly reduced in EOC tissues and cell lines relative to paired non-cancerous tissues and IOSE cell line, respectively. They also showed that miR-184 overexpression in transfected cells inhibited EOC cell proliferation and suppressed inflammation by providing lower levels of several cytokines, such as the tumor necrosis factor alpha (TNF-α), and interleukins IL-6, IL-8, and IL-10. In vitro induction of apoptosis was detected by analysis of apoptosis-related genes, which showed decrease in the expression of Bcl-2 and increased expression of Bax and caspase-3 activity in EOC cells compared with respective controls. Notably, investigation of survival among EOC patients using Kaplan-Meier survival analysis revealed worse overall survival compared to those with high expression of this miRNA. Additionally, Cox regression multivariate analysis suggested miR-184 as an independent biomarker for EOC patients. These findings suggest miR-184 as a prognostic biomarker for OC patients along with its significance in the disease pathogenesis [156].

## Other cancers

In a number of other cancers dysregulation of miR-184 as well as its significance in the carcinogenesis is documented. For instance, in clear cell renal cell carcinoma (ccRCC), miR-184-5p is reported with downregulation in A-498 and 786-O cell lines and its overexpression showed a tumor suppressor role suppressing the proliferation, and invasion and induction of apoptosis by directly targeting the NUS1 dehydrodolichyl diphosphate synthase subunit (NUS1) [157]. In melanoma, among a number of candidate miRNAs with potential role in regulating the aggressive features of cancer cells, miR-184 was shown to markedly inhibit the invasive capability and tube formation activity of HAG cells [158]. The tumor-suppressing role of miR-184 is also documented in human central nervous system lymphoma (HCNSL) cells by inhibiting the cell survival and invasion of HCNSL cells via regulating the PI3K/Akt signaling pathway [159]. Although an increasing number of ncRNAs are studied in retinoblastoma [160], miR-184 is documented in a single study with a tumor suppressing function, enhancing chemosensitivity through targeting SLC7A5 [161].

## Clinical significance of miR-184 in human cancers

A biomarker is defined as a biological compound, which is detectable in body specimens and represents a specific physiologic process, a pathologic condition/outcome, or response to a pharmacologic intervention or exposure [162–164]. In a general classification, tumor biomarkers are divided into prognostic, used for prediction of survival and response to treatment, and diagnostic with potentials in differentiating cancer patients from healthy individuals or help diagnose a specific type of cancer [165]. Aberrant expression of ncRNAs, including miRNAs, may be used as a potential in the prediction of prognosis and diagnosis of cancer patients [166–170]. Showing a correlation with worse clinicopathological features as well as shorter survival rates among cancer patients, low levels of miR-184 may act as a prognostic biomarker for some cancers (see Table 1). In glioma, for example, miR-184 expression is shown to negatively correlate with survival time of those patients [109]. Moreover, miR-184 has shown acceptable diagnostic performance in the area under curve (AUC), Cox regression as well as univariate and multivariate analyses indicating that it may be efficient in diagnosis of cancer patients. For instance, survival analysis using Kaplan–Meier plot in EOC patients showed that patients with low expression levels of miR-184 had worse overall survival compared to those with high expression. Further analyses using Cox regression multivariate analysis suggested miR-184 as an independent biomarker for EOC patients [156]. Beyond the prognostic/diagnostic performance of miR-184, its putative roles in the carcinogenesis of various human cancers and contributions to enhanced chemosensitivity indicate its potentials in cancer therapeutics [161]. miRNA-targeted therapy is suggested as a therapeutic strategy for human cancers [19]. The development of novel approaches for effective and safe delivery of miRNAs to restore their tumor suppressor function in targeted tumor tissues is a research hot spot for cancer scientists. Delivery approaches including viral vectors, nanoparticles, and extracellular vesicles (EVs) have been tested for miRNA-targeted therapy [171]. Owing to their potential in loading various biological compounds, such as protein, miRNA, and DNA as well as drugs [172], EVs are being increasingly studied as ideal drug delivery vehicles for cancer therapeutics [173]. In cell experiments, viral vectors are mainly used for the delivery of miRNA constructs to the target cells for whom results mainly demonstrated that exogenous expression of miR-184 may suppress the malignant transformation of cancer cells and thus, suggest a therapeutic potential (Table 1).

## Concluding remarks

miRNAs play crucial roles in posttranscriptional modulation of gene expression and regulate various biological functions and critical processes. Accordingly, their aberrant expression is associated with many pathologic conditions, particularly cancer. miR-184 is a well-studied miRNA, which is mainly reported to play a tumor suppressor role by inhibiting the malignant phenotype of cancer cells, although some controversial reports claim opposite effects. Affecting signaling pathways, oncogenic factors or apoptotic proteins are some discovered mechanisms through which miR-184 might function as a tumor suppressor. The aberrant expression of miR-184 is frequently reported in tumor tissues retrieved from cancer patients and an association between its expression levels and clinicopathological characteristics among cancer patients suggests it is a reliable prognostic biomarker. Moreover, miR-184 might be employed for the detection of cancer patients as a potential diagnostic biomarker. Additionally, experiments using exogenous miR-184 mainly support its role as a tumor suppressor inhibiting tumor growth and metastasis in vitro and in vivo highlighting its significance as a therapeutic target for cancer patients.

## Data Availability

No data were used for the manuscript
